# A proposal for a new temperature-corrected formula for the oxygen content of blood

**DOI:** 10.1186/s40981-020-00368-x

**Published:** 2020-08-12

**Authors:** Kiichi Hirota, Miyahiko Murata, Koh Shingu

**Affiliations:** 1 Department of Human Stress Response Science, Institute of Biomedical Science, Kansai Medical University, Hirakata, Osaka 573-1010 Japan; 2 Department of Medical Care and Welfare Engineering, School of Industrial and Welfare Engineering, Tokai University, Kumamoto, Kumamoto 862-8652 Japan; 3 Department of Anesthesiology, Osaka Red Cross Hospital, Osaka, Osaka 543-8555 Japan

To the Editor,

Oxygen (O_2_) is essential for normal aerobic metabolism in mammals. O_2_ delivery is the process of transport of oxygenated blood from the alveolar capillaries to the tissues and is dependent on two factors: arterial O_2_ content (CaO_2_) and cardiac output. The carriage in the blood occurs as O_2_ bound to hemoglobin and O_2_ dissolved in the plasma and is expressed as the sum of these components with the following equation: CaO_2_ = 1.39 × Hb (g/dl) × SaO_2_ + 0.0031 × PaO_2_ (mmHg) (1). CaO_2_ is the milliliters of O_2_ per 100 mL of blood, SaO_2_ is the fraction of hemoglobin (Hb) that is saturated with O_2_, the O_2_-combining capacity of Hb is 1.39 mL of O_2_ per gram of Hb, Hb is grams of Hb per 100 mL of blood, PaO_2_ is the O_2_ tension, and solubility of O_2_ in plasma is 0.003 mL of O_2_ per 100 mL plasma for each mm Hg of PaO_2_ [1, 2]. The pressure is 1 atm (101.325 kPa). However, the temperature is not clearly specified. In fact, the coefficient of 1.39 in the previous item of the equation was obtained under the standard condition of 0 °C (273 K). It is obtained as 22.4 (volume of 1 mol of gas at 273 K) × 4 (molecular number bound to 1 molecule of hemoglobin)/64,458 (molecular weight of hemoglobin)/1000 (correction coefficient) [3, 4]. In the case of 37 °C (310 K), the coefficient should be 1.58 due to Boyle-Charles law or combined gas law (273 + 37)/273 × 1.39. On the contrary, the coefficient 0.0031 from the last item of the equation is derived from evidence on O_2_ solubility at 37 °C (310 K). It is obtained as 0.0239 (Bunsen absorption coefficient α of oxygen at 37 °C) × 100/760 [5]. These items from the same equation are derived at different temperatures. Although equation (1) is established and is described in almost all published textbooks, the equation should be revised so that it is more clinically relevant. In lieu of equation (1), we propose a new temperature correction formula: CaO_2_ (ml/dl) = 1.58 × Hb (g/dl) × SaO_2_ + 0.0031 × PaO_2_ (mmHg) at 37 °C and 1 atm. Lack of experimental results is one of limitations of our proposal, and we need to confirm them in clinical settings.

## Data Availability

Not applicable
