# Correction to: A proposal for a new temperature-corrected formula for the oxygen content of blood

**DOI:** 10.1186/s40981-021-00415-1

**Published:** 2021-01-26

**Authors:** Kiichi Hirota, Miyahiko Murata, Koh Shingu

**Affiliations:** 1 Department of Human Stress Response Science, Institute of Biomedical Science, Kansai Medical University, Hirakata, Osaka, 573-1010 Japan; 2 Department of Medical Care and Welfare Engineering, School of Industrial and Welfare Engineering, Tokai University, Kumamoto, Kumamoto, 862-8652 Japan; 3 Department of Anesthesiology, Osaka Red Cross Hospital, Osaka, Osaka, 543-8555 Japan

**Correction to: JA Clin Rep 6, 62 (2020)**

**https://doi.org/10.1186/s40981-020-00368-x**

Following the publication of the original article [1], the authors recognized that they need to revise some of their discussion and the explanation is provided as follows.

We thought that the coefficient 0.0031 was the Bunsen coefficient of oxygen to water measured at 37 °C, but it turned out that the Bunsen coefficient was the coefficient obtained by converting the value measured at the relevant temperature to 0 °C under 1 atm conditions.

As a result, we determined that the formula calculated below is appropriate when temperature correction is performed at 37 °C.

From:

In lieu of Eq. (1), we propose a new temperature correction formula: CaO2 (ml/dl) = 1.58 × Hb (g/dl) × SaO2 + 0.0031 × PaO2 (mmHg) at 37 °C and 1 atm.

To:

In lieu of Eq. (1), we propose a new temperature correction formula: CaO_2_ (ml/dl) = 1.58 × Hb (g/dl) × SaO_2_ + 0.0035 × PaO_2_ (mmHg) at 37 °C and 1 atm.

The authors apologise for this error.
